# Creating opportunities for patient participation in managing medications across transitions of care through formal and informal modes of communication

**DOI:** 10.1111/hex.13524

**Published:** 2022-05-27

**Authors:** Guncag Ozavci, Tracey Bucknall, Robyn Woodward‐Kron, Carmel Hughes, Christine Jorm, Elizabeth Manias

**Affiliations:** ^1^ Alfred Health Melbourne Victoria Australia; ^2^ The School of Nursing and Midwifery, Centre for Quality and Patient Safety Research, Institute for Health Transformation Deakin University Burwood Victoria Australia; ^3^ Department of Medical Education, Melbourne Medical School The University of Melbourne Melbourne Victoria Australia; ^4^ School of Pharmacy Queen's University Belfast Belfast Northern Ireland; ^5^ School of Public Health The University of Sydney Sydney New South Wales Australia

**Keywords:** aged, communication, continuity of patient care, health personnel, medication therapy management, patient participation, patient transfer

## Abstract

**Background:**

Communicating about medications across transitions of care is important in older patients who frequently move between health care settings. While there is increasing interest in understanding patient communication across transitions of care, little is known about older patients' involvement in formal and informal modes of communication regarding managing medications.

**Objective:**

The aim of this paper was to explore how older patients participated in managing their medications across transitions of care through formal and informal modes of communication.

**Methods:**

The study was conducted across two metropolitan hospitals: an acute hospital and a geriatric rehabilitation hospital in metropolitan Melbourne, Australia. A focused ethnographic design was used involving semi‐structured interviews (*n* = 50), observations (203 h) and individual interviews or focus groups (*n* = 25). Following thematic analysis, data were analysed using Fairclough's Critical Discourse Analysis.

**Results:**

Data analysis revealed two major discursive practices, which comprised of an interplay between formal and informal communication and environmental influences on formal and informal communication. Self‐created patient notes were used by older patients to initiate informal discussion with health professionals about medication decisions, which challenged traditional unequal power relations between health professionals and patients. Formal prompts on electronic medication administration records facilitated the continuous information discourse about patients' medications across transitions of care and encouraged health professionals to seek out older patients' preferences through informal bedside interactions. Environmental influences on communication comprised health professionals' physical movements across private and public spaces in the ward, their distance from older patients at the bedside and utilization of the computer systems during patient encounters.

**Conclusion:**

Older patients' self‐created medication notes enabled them to take on a more active role in formal and informal medication communication across transitions of care. Older patients and family members did not have continuous access to information about medication changes during their hospital stay and systems often failed to address older patients' key concerns about their medications, which hindered their active involvement in formal and informal communication.

**Patient or Public Contribution:**

Older adults, family members and health professionals volunteered to be interviewed and observed.

## 1 INTRODUCTION

Communicating about medications across transitions of care can be problematic for older patients since they are likely to have complex health conditions and multiple changes to their medications as they move between health care settings.^1^, ^2^ Transitions of care refer to patients' movements between different locations including hospitals, residential care facilities or patients' homes. They also involve the communication processes in preparing for patients' movements between different levels of care, between different care teams and clinicians within the same location and include patients' interactions with various health professionals as their needs alter during the course of an illness.^3^ Transitions of care communications do not only cover communications occurring at the point of admission or discharge but also encompass intra‐ and interhospital interactions taking place at different stages in the care pathway between admission and discharge, particularly any interactions about changes in patients' clinical goals, care needs and medications that may impact the planning processes of patients' movements. The World Health Organization indicated that about 26% of hospital readmissions involving older patients are associated with medication incidents, frequently due to omissions of medications and failure to communicate medication changes across transitions of care.^4^

Communication comprises exchanging and understanding medication information by using written, electronic, verbal and nonverbal means.^5^ Formal modes of communication involve planned communication events including ward rounds, clinical handovers, team meetings, family meetings, admission and discharge medication counselling, as well as health professionals' documentation of patients' progress, treatment goals and medication regimens in medical records. Informal modes of communication refer to opportunistic, unplanned, spontaneous, unstructured interactions that can take place at any time in different locations such as at the bedside, in corridors and in office spaces, and also involve self‐initiated communication aids including handwritten notes or reminder scribbles.^6^

Older patients' participation in medication communication can differ depending on the structure and the context of communication events that they are involved in as well as the severity of their cognitive impairment during hospitalization.^7^, ^8^ Although involving older patients with cognitive impairment in formal interactions is challenging for health professionals, a few strategies have been found to be helpful for patients with mild cognitive impairment such as simplifying decisions, holding the discussions in a quiet environment, using teach‐back methods and using printed tools including decision aids.^9^ Previous studies also indicated lack of involvement of older patients in formal communication encounters during ward rounds where discourses were shaped by organizational culture, clinician behaviours and entrenched power relations between patient–provider relationships.^3^, ^10^, ^11^, ^12^, ^13^ For example, tensions between patient‐centred discourses and organizational discourses in medical or nursing education have been reported where senior doctors focused more on meeting the educational needs of junior medical staff or where nursing educators focused on memorizing isolated facts about medications than involving patients in decision‐making processes.^14^, ^15^ Studies of formal ward rounds have revealed inconsistent engagement with patients, patients' experiences of health professional dominance and the exercise of hierarchical power by controlling medication decisions as well as the spaces where communication occurred.^10^, ^12^ Furthermore, organizational factors, such as competing demands of health professionals, staff workloads, temporal and spatial challenges,^16^ or inaccurate information transfer between health professionals and settings have been identified as hindrances to collaborative conversations between health professionals and patients during formal encounters upon patient admission and at hospital discharge.^17^ Temporal challenges included lack of availability of patients or family members at a time that coincided with health professionals' availability, whereas environmental and spatial challenges included barriers to communication due to distance of health professionals from patients' bedside.^18^ Notably, there has been a lack of focus on informal interactions between patients and health professionals concerning medication information^19^ and on patients' proactive communication about their needs and goals as they move across settings.^16^ Previous research on formal and informal communication has predominantly focused on interactions more generally between health professionals and patients without considering medication communication in the context of transitions of care.

The aim of this paper was to explore how older patients participated in managing their medications across transitions of care through formal and informal modes of communication.

## 2 METHODS

### 2.1 Study design

A focused ethnographic design using multiple methods was adopted to explore communication processes between older patients, family members and health professionals in relation to managing medications across transitions of care.

### 2.2 Setting and study participants

This study took place in two metropolitan hospitals in Melbourne, Australia: a 500‐bed tertiary teaching hospital and a 150‐bed geriatric rehabilitation facility. Data collection was conducted in a general medicine ward and two medical wards specialized in infectious diseases and general respiratory in the teaching hospital, and in three aged care and two rehabilitation wards in the geriatric rehabilitation facility.

Participants comprised older patients, family members and health professionals. Inclusion criteria for patients were those aged 65 years or older on admission. We chose this age cut‐off because 65+ has been conventionally used to designate an older person in the literature.^20^, ^21^ Older patients with severe cognitive impairment were excluded. Researchers screened all potential patients with nurse unit managers of designated wards to make sure that they were cognitively capable of consenting before approaching them at the bedside. Every older patient had a Glasgow Coma Scale (GCS) score documented by ward nurses in the observation charts within patients' medical records. This score was used to complement other means of determining patient eligibility to participate, which included discussions with the nurse‐in‐charge, patients and family members. Older patients who had a GCS score lower than 14 were not included in the study. Inclusion criteria for family members were any individuals who were associated with older patients admitted to hospital. Inclusion criteria for health professionals were registered nurses, pharmacists and doctors employed for at least 1 week at the study settings.

Two researchers from nursing and pharmacy backgrounds undertook data collection. In consultation with nurse unit managers and bedside nurses, researchers initially viewed patient lists of each study ward to determine the potential eligibility of patients. After providing written and verbal information about the project at the bedside, researchers obtained informed consent from older patients. Purposive sampling was adopted to ensure that the older patients represented various age categories including youngest‐old (65–74), middle‐old (74–84) and oldest‐old (85 years or over). Older patients were approached at the bedside at a time that was convenient for them. We approached 44 older patients in acute care settings and 34 patients in subacute care settings. Older patients were given unlimited opportunity to decide whether they wished to take part. Of those who were approached in acute care settings, 26 patients accepted and 18 patients declined, whereas, 24 patients accepted and 10 patients declined in subacute care settings. Health professionals were also recruited using purposive sampling taking into consideration their discipline, level and the length of their experience to ensure a variety of professional backgrounds. Purposive sampling was conducted by identifying specific characteristics in older patients and health professionals and ensuring that all characteristics were covered before the recruitment. During the recruitment of participants, information about the study was provided through participant information sheets and a verbal set script summarizing the information that was included in the participant information sheet. Participants were provided sufficient time to read the study information and consent form and ask questions. The time given to patients to consider whether or not to take part ranged from a few minutes to a couple of days depending on patients' availability to read the participant information and consent forms.

### 2.3 Data collection

Data collection comprised semi‐structured interviews, unstructured observations and individual or focus groups at both hospital sites from April 2018 to October 2019. Researchers conducted semi‐structured interviews with older patients and family members, who could communicate in English, by using open‐ended questions, focusing on obtaining older patients' perspectives on medication communication at transitions of care. Questions involved how health professionals talked to older patients about their medications on admission, during their hospital stay and on discharge, what things were important to discuss about their medications as they moved between settings and whether patients' medications were changed and if and how those changes were communicated to them by health professionals (File S1). The duration of each interview was approximately 20 min.

Observations were undertaken of health professionals in their interactions with older patients and families at different times to cover all working shifts. Credibility was assured through investigator triangulation. The research team involved six investigators and all investigators were involved in critical interpretation of the data. Observations were audio‐recorded and conducted by two researchers separately. After each observation, researchers made field notes documenting the length and sequence of events, activities that participants carried out, physical layout of the settings, emotions expressed and participants' actions. Since researchers shadowed the health professionals during their shifts on dedicated wards, the observations were usually captured at times between patients' admission to the hospital, transfers between wards in the hospital and discharge. In addition, researchers managed to capture a considerable number of interactions that occurred between patients and health professionals at the exact point of discharge. Besides, researchers collected data from medical records to make sense of the interactions captured during observations. Data collected from medical records involved medications prescribed at hospital, medications changed, started or withheld during a previous admission, suspected nonadherence to medications, medication allergies and goals of care relating to the medication regimen.

Lastly, individual interviews or focus groups were conducted with older patients and family members to obtain their reflections on the issues that were identified via interviews and observations. Researchers organized individual interviews or focus groups as face‐to‐face sessions. Individual interviews or focus groups were undertaken depending on the availability of older patients and family members. In the instances where family members were available, a focus group was undertaken, which usually comprised an older patient and one or two family members at the bedside. Researchers developed a focus group schedule including the key issues that were obtained through the preliminary analysis of interviews and observations. Topics included in focus group schedule included patients' experiences talking with different health professionals about their regular medications across transitions of care, if and how health professionals could help them to manage changes to their medications and how they preferred health professionals to talk about their medications when they counsel them on admission or at discharge. All data collected at interviews, observations and focus groups were audio‐recorded and transcribed verbatim. Data collection ceased after repeated patterns of information were obtained in each ward setting.

### 2.4 Data analysis

Initially, verbatim transcriptions of data were read and reread to increase familiarity. First, to explore key characteristics of issues, the research team coded a selection of transcriptions. The first selection comprised five different transcriptions and all researchers independently coded those transcriptions inductively. After all codes were compared and contrasted and any discrepancies were resolved amongst the research team, a thematic coding framework was constructed to be applied to the remaining transcripts. All transcriptions and the coding framework were transferred into NVivo 12 (QSR Melbourne), and the remaining data were coded by three researchers independently. Codes were clustered into larger groupings and into themes.^22^ Data analysis was discussed at fortnightly research team meetings.

The Critical Discourse Analysis (CDA) developed by Fairclough was used to conduct a complementary discourse analysis of the data. According to a Faircloughian approach to CDA, discourse is associated with language use as a form of social practice. CDA is the synthesis of theoretical positions that provide opportunities to conduct a critical examination of the relations between power inequalities and language, discourse and social contexts and language and ideology. Given that a focused ethnographic design comprises exploring social phenomena and shared experiences within a specific culture, using CDA was a suitable method of analysis that allowed the researchers to explore overarching ideologies, roles, social relations, unspoken rules of discourses relating to whose voices were dominant or marginalized and common‐sense assumptions affecting health professionals' everyday social practices. The CDA approach helped us to explore the data at three different dimensions including the text level, the discursive practice level and the social practice level.^23^ The text level involved examining the structure of the communication encounters, the aspects of the language that people used and the content that was prioritized. At this level, we also analysed language devices including the use of humour, modality, back‐channelling and hedging according to their broad definitions. The discursive practice level explored how discourses were produced in various ways in specific social contexts. At this level, researchers focused on exploring power relations between participants, roles and professional status of participants, the way they were positioned in the interaction and times and places that were relevant to conversations. Finally, at the social practice level, researchers examined the influences of wider organizational and ideological challenges on the discursive practices in health care settings. At the social practice level, the focus was on examining whether the communication challenged or empowered the existing status quo or whether discursive practices were expressed in contemporary or innovative ways. The research team including six members developed a codebook comprising the data analysis questions mapped under each level of CDA to guide the data analysis process. These research questions were derived from the CDA through discussions amongst team members considering past studies investigating medication communication between health professionals and patients and the objectives of the research.^24^, ^25^ Three researchers analysed the data independently according to the codebook and discrepancies were discussed and resolved during regular research team meetings.

### 2.5 Ethics

This study was approved by the university committee, Deakin University Human Research Ethics Committee (DUHREC) 2018‐067 and the hospital ethics committee, HREC 212/17 (the full name of the ethics committee is not disclosed in accordance with requirements of ethics approval and to protect the identity of the participants). Researchers used pseudonyms when transcribing and analysing the data to maintain the confidentiality. Verbal and written consent was obtained from patients to conduct individual interviews and focus groups and to collect the data from medical records. Informed consent was also obtained for the use of audiovisual media such as photos. For observations, researchers attempted to obtain written consent from health professionals and older patients. If, after explaining the project, the older patient wished to participate, but was unable to read the participant information and consent form due to visual problems, general tiredness and lack of desire to read, or was unable sign the consent form due to poor hand grip as a result of frailty, a process of verbal consent was followed thereafter. This process of obtaining verbal consent was approved by the ethics committees. Any identifiable information, such as patients' names or name of the hospital, was removed at the time of data collection. The confidentiality of data collected from medical records was ensured by associating these data with the patient pseudonyms instead of using real names during data collection processes. The data collected from medical records were stored on an online‐secured storage drive of the University. To identify eligible patients from patient lists, researchers obtained permission from head medical consultants and nurse unit managers of each study unit to access these patient lists. This process was also approved by the ethics committees.

## 3 RESULTS

In all, 50 older patients participated in semi‐structured interviews. In addition, 25 focus groups were conducted across both hospitals. If family members were not available for a focus group, individual interviews with older patients were conducted instead. Twenty older patients and 13 family members participated in focus groups or individual interviews if focus groups were not possible. Observations were conducted with 29 health professionals and 111 patients for a total of 203 h. The demographic characteristics of the participants are shown in Table 1. Two major discursive practices were interpreted through the data: An interplay between informal and formal communication and environmental influences on formal and informal communication.

**Table 1:** Study characteristics.

Characteristics of older patients who participated in interviews and focus groups	*n* (%)
Age	
Youngest‐old (65–74)	38 (54.3%)
Middle‐old (74–84)	18 (25.7%)
Oldest‐old (85 years and over)	14 (20.0%)
Gender	
Female	41 (58.6%)
Male	29 (41.4%)
Country of birth	
Australian	46 (65.7%)
England	6 (8.6%)
Germany	3 (4.3%)
Scotland	2 (2.9%)
South Africa	2 (2.9%)
Ireland	2 (2.9%)
Czech Republic	1 (1.4%)
Greece	1 (1.4%)
Italy	1 (1.4%)
Romania	1 (1.4%)
Ukraine	1 (1.4%)
USA	1 (1.4%)
Indonesia	1 (1.4%)
Peru	1 (1.4%)
Total	70
Prehospital admission medication	
Self	55 (78.6%)
Family member	8 (11.3%)
Dose administration aids	6 (8.6%)
Home nurse	1 (1.4%)
Total	70

Study sites (subacute & acute)	Interviews (*n* = 50)	Focus groups (*n* = 20)
Subacute1 (aged care)	10	3
Subacute2 (aged care)	11	4
Subacute4 (rehabilitation)	3	5
*Total (subacute)*	*24*	*12*
WardMed1 (Infectious diseases, neurology, stroke)	8	0
WardMed2 (General Medicine)	10	5
WardMed3 (General Respiratory)	8	3
*Total (acute)*	*26*	*8*
*Overall interviews*	*50*	*20*

**Observations conducted with health professionals**	**Health professionals (*n*** = **29)**	**Hours (203.95)**
Nurses		
Subacute	8	49.2
Acute	12	88.0
Pharmacists		
Subacute	3	21.3
Acute	2	12.45
Doctors		
Subacute	2	11.5
Acute	2	21.45

### 3.1 Interplay between informal and formal communication

Communication via formal and informal modes flowed backwards and forwards in health care settings, which created opportunities for older patients' participation in decision‐making processes. Self‐created patient notes and the prompts on medical records enabled patient involvement in medication changes across transitions of care.

#### 3.1.1 Self‐created patient notes

Older patients who were interested in managing their medications independently were inclined to create written notes about their medications to use as communication aids during their hospital stay, and when they moved between settings. In all, 20% of older patients who participated in the study (*n* = 36) were observed to create medication notes. These patients often had multiple comorbidities, independently managed medications or used a dose administration aid at home. They also demonstrated self‐motivation to learn about changes to their medications and side effects. Older patients were concerned that ward rounds usually occurred at unpredictable times and these notes provided them with tangible resources, reminding them to ask questions during ward rounds. These notes included hand‐written lists about their regular medications, changes made to medications commenced in hospital or reminder scraps for issues that they wanted to raise during health professionals' bedside visits. During interviews, a 67‐year‐old patient emphasized the importance of keeping a written history of regular medications to be able to answer queries raised by doctors after hospitalization:

It's important to let them all know what exactly I'm taking and the dosage … and if I can't remember it I've written it all down in a history of my medications which has got a history of my last procedures with my doctors. Because, you know, you can't remember. They come along to you and say, ‘Right, when did you have your last operation and what was it?’ So, I've written it all down and keep that.

Interviews and observations also revealed that older patients commonly created written medication notes and sometimes used these notes as reminders to query medication changes from previous care settings during bedside discussions with ward pharmacists to clarify the rationale behind those changes. These patients seemed to be heavily reliant on their previous medication regimens and felt uncomfortable with the changes made to their regular medications at the transferring hospital. For instance, an 81‐year‐old patient talked to the pharmacist about a conflict that he had with doctors regarding the change that they made to his regular insulin regimen: ‘The insulin, the morning dose was ahmmm… I have got it written down actually, because we had an argument. I said that (referring to the morning dose) should be at least 24 not twelve!’ Further, in the situations where health professionals did not attempt to involve older patients in the formal interactions due to their cognitive decline, family members became vocal in decision‐making processes and took on a proactive role in keeping the records of medications on behalf of their relatives.

In the following observation, the nurse was administering morning medications and a 70‐year‐old patient was unwilling to take aperient Movicol® (macrogol) since he believed that the timing was inappropriate. He created notes to remind himself to discuss the timing of medication doses with the doctors (Table 2).

**Table 2:** The communication between the nurse and the patient during the medication administration process.

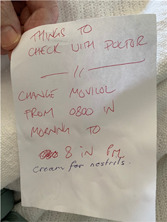	*Nurse*: Ok. Did you end up having a chat with the doctors yesterday about that?
	*Patient*: Haven't seen the doctors, they're scared to talk.
	*Nurse*: They must be! … When you see the doctors, tell them exactly that (referring his notes). They might be happy to switch it to the night time. But for the moment we want you to have a good movement, you didn't have a great one yesterday. (talks about bowel movement).
	*Patient*: That's why, I want to take it at night. It works in the morning.
	*Nurse to the researcher*: He has written notes for himself to ask the doctors when the doctors come around. (Researcher reads patient's note)… That way he doesn't forget to ask them. Because often they'll start talking about something else and you'll forget‐ you forget what you wanted to ask.
*Patient*: Yes. That's exactly right.	
*Excerpt code: RN13_Subacute2_20190917_Interaction4_Pt105	

The patient was unhappy about the lack of availability of doctors since there was no opportunity to ask for the timing of administration of Movicol® to be moved from morning to night. The patient was also frustrated by his inability to remember to relay his complaint during ward rounds, so had taken to writing his questions on a piece of paper as a reminder. The nurse supported the patient writing reminder notes, emphasizing the challenge of recalling the medication‐related questions during ward rounds since the flow of the conversation was usually steered by doctors. When questioned at a later time, the patient confirmed that his notes helped him to ask questions and provide suggestions about changing the administration time of his laxative, which indicated that the older patient's interest was eventually being served since he positioned himself as an active agent in urging doctors to change the administration time of the medication. Self‐created patient notes prompted the patient to initiate informal discussion to impact medication decisions, which eventually played a role in shifting the balance of power from doctors to the patient during ward round interactions.

In the following interview excerpt, an 81‐year‐old patient reported the importance of having hand‐written medication lists that she prepared and including notes in this list. This was the patient's second admission to the same hospital and she was referring to her own medication notes that she took during her previous hospitalization:

So I think I can't see any other way other than verbally and then in writing…I wrote right back to when I first came in here on the 17th March (Patient's previous hospitalisation date). So that I could answer questions … I've written myself a note ‘*Why was I taken off Slow K? My potassium is very low now and I've had a drip for twelve hours which hopefully has replaced what has been lost*’. I wrote that because I want to ask that question. (Int_Pt6_WardMed1)

The patient regarded the written notes as a necessary communication aid in addition to health verbal interactions with health professionals. The patient structured her notes using questions so that she was able to raise her concerns about changes to her potassium medication to health professionals during ward rounds. The rationale behind the cessation of potassium was not communicated to her before her previous discharge, which encouraged her to prepare her notes in this way:

Well I did ask about why they would have taken me off the slow K which is the potassium when I originally came into hospital, but no one has an answer to that … And they didn't tell me, I'd like them to have said, ‘Look, your, what's a name's too high, so we're taking you off it’. (Int_Pt6_WardMed1)

The patient took on a proactive role in the initiation of future communication because her self‐written notes served as an aide‐memoire for her medication concerns that needed to be clarified with health professionals. On this occasion, the patient used her notes as a last resort to attract health professionals' attention when the important medication information had not been provided by them during formal interactions across care transitions. However, there were occasions where not all patients were motivated to keep medication notes to assist with participation during ward rounds. Instead, they trusted that information about medication changes made during their hospital stay would be eventually conveyed to their GP so that these changes could be discussed following discharge. During a focus group interview, a 76‐year‐old patient reported:

When I go to the doctor, I expect that all my details from here will have gone to him. So that when he opens his computer, he's got all my details there and I can talk about things and medication changes. (Pt20_FG20_Subacute2)

#### 3.1.2 Prompts that encourage engagement with older patients

Prompts referred to health professionals' formal notes, comments and reminders about patients' medications or small templates indicating patients' medication management on the electronic medication administration record (eMAR). These prompts were prepared within a discourse of efficiency to facilitate interaction amongst health professionals throughout patients' transition journey; however, they encouraged health professionals to have brief, face‐to‐face interactions with patients about specific medications or organizing counselling sessions about their medications. Prompts were predominantly used by pharmacists as a conduit to address adherence issues, for the purpose of addressing the use of benzodiazepines, ensuring appropriate dose of insulin and enabling accurate medication‐taking of statins. These older patients had demonstrated the discourse of self‐determination by following their own medication decisions instead of accommodating health professionals' medication advice. In these cases, tensions arose between pharmacists' need to address medication safety goals of care and patients' desire to manage their medications in their own way. The informal bedside communications conducted by pharmacists following their observations of these prompts on the eMAR enabled tensions to be examined before patients' hospital discharge.

**Figure 1:**
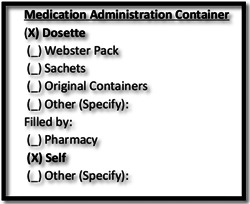
A template demonstrating patients' self‐medication administration on electronic medication administration record.

In the following example, the pharmacist was locating the information on the eMAR about medications of an 80‐year‐old patient who had recently undergone heart transplant surgery. The pharmacist was thinking aloud to facilitate the training process while she was with the intern pharmacist during her shift (Figure 1):

Charted.Charted.stopped.stopped.He doesn't have any Mag (Magnesium). He needs Mag (Magnesium) and [nicotine that he might]. Soo…not charted.charted. Rosuvastatin's not charted.Soo…He usually has a Dosette box, which he packs himself. Then he goes to his pharmacy and outpatient pharmacy for his rejection meds. He has not had a stroke anything like affecting his brain. So, I probably would imagine he'd be able to continue self‐management of meds in the same way. But we'll check in with him and make sure that's the case. (Pharm5_Obs_Subacute5_Interaction9)

During the observation, the pharmacist noticed some changes prescribed by doctors since the patient's admission to the hospital. In addition to the changes to his medications, the pharmacists viewed a small template under ‘Medication Administration Container’ specifying that the patient managed his medications by himself at home by using dose administration aids. This prompted the pharmacist to engage with the patient through an informal bedside conversation to confirm if he was still going to be able to manage his medication by using the Dosette box after discharge.

**Figure 2:**
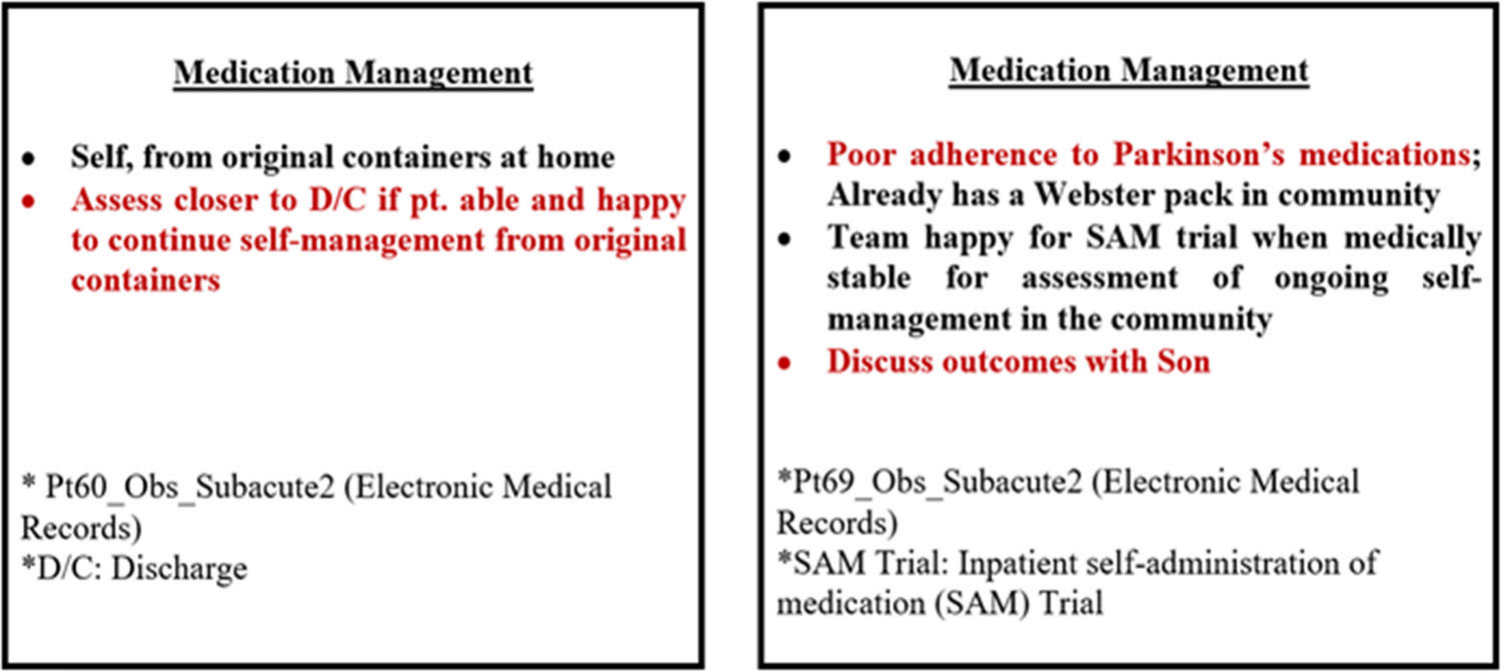
Inclusive statements under ‘Medication Management’ sections on electronic medication administration record.

**Figure 3:**
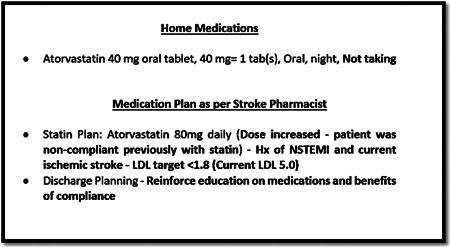
Admission notes and discharge medication plan on eMAR. *Note*: Hx of NSTEMI and LDL. eMAR, electronic medication administration record; LDL, low‐density lipoprotein; NSTEMI, history of non‐ST‐elevation myocardial infarction.

Some prompts were made up of statements that are inclusive of patients and carers in medication decisions, which triggered pharmacists' engagement with older patients and family members preparing for discharge to investigate patients' capability and preferences in managing their medications. Upon noticing that pharmacists visited them at the bedside to seek their opinions on medications, older patients felt included in decision‐making and encouraged to express their preferences. Figure 2 shows two separate older patients' medical records displaying these inclusive prompts, which constituted tensions between discourse of self‐medication management and discourse of reliance on family members or dose administrations aids in managing their medications. The informal bedside interaction with older patients enabled the pharmacist to examine these tensions and check if the patient would still feel comfortable in managing medications independently after discharge (Figure 2):

Similarly, in the following observation, the pharmacist was viewing a 69‐year‐old patient's medications on the eMAR and looking at ‘Home Medications’ recorded after the patient's admission and the stroke pharmacist's notes under the ‘Medication Plan’ section at the same time. The pharmacist checked the notes about home medications and the stroke pharmacist's discharge medication plan and noticed that the patient was not adherent to atorvastatin (lipid‐lowering agent) at home and needed to be educated about it (Figure 3):

Different notes indicated the patient's history of lack of adherence with his statin treatment (Figure 3), which propelled the pharmacist to discuss the importance of his statin treatment. The pharmacist was aware that the patient's wife was the main carer looking after the patient's medications at home; therefore, she decided to include the wife in the discussion of the patient's medications (Table 3).

**Table 3:** Pharmacist education on the importance of taking atorvastatin

*Pharmacist*: Atorvastatin, which is also known as Lipitor®.
*Wife*: Lipitor®. Yeah I know Lipitor®.
*Pharmacist*: And he was on 40 milligrams, now after as someone has had a stroke and also had a heart attack, we try to maximise their statin.
*Wife*: Ok. So 80.
*Pharmacist*: 80. And we would do that because not just for cholesterol and lipids which is what it's traditionally used for, but also there might be a site on that blood vessel which has a plaque which may have contributed to the stroke. So this helps stabilise that plaque to prevent further strokes. And there's good evidence to show that it does that. I know‐
*Wife*: That's right.
*Pharmacist*: And yeah I know in the media there's a bit of‐
*Wife*: Bit of negative?
*Pharmacist*: Yeah.
*Wife*: Yeah.
*Pharmacist*: But not‐ I think that's more to do with primary prevention but this is for secondary prevention and there is a lot of good ‐ strong evidence to support that.
*Wife*: That it helps.
*Pharmacist*: Yes.
*Excerpt code: Pharm5_Obs_Subacute5_Interaction6

The communication occurred with the patient's wife away from the bedside in the communal area of the ward since the pharmacist found that the bedside area was noisy. However, the patient was missing in this interaction, which demonstrated how environmental struggles within bedside spaces can inhibit the patient's involvement in discussions. The pharmacist adopted persuasive discourse to challenge misconceptions against the use of statins by providing evidence for their benefits. The pharmacist's use of medical jargon such as ‘plaque’, ‘primary’ and ‘secondary intervention’ could have contributed to the wife's difficulties in understanding what these concepts meant. The pharmacist made no attempt to check back on her interpretation of these concepts. The use of hedging by the pharmacist appeared to be a communicative strategy to express tentativeness in her statements about statin treatment (i.e., ‘I think’, ‘we would’, ‘there might be’, ‘may have’) and also to gain some protection from possible criticism (‘we would’, ‘I think’).

### 3.2 Environmental influences impacting the flow of informal and formal medication communication

Environment factors influenced older patients' engagement in both informal and formal interactions about medication decision‐making. These factors consisted of health professionals' physical positioning in the bedside environment or across private and public spaces at hospital and the utilization of computer systems at the bedside during formal and informal medication communication. Mobile computers facilitated health professionals' access to patients' electronic medical records at the point of care.

#### 3.2.1 Physical positioning of health professionals

Health professionals' use of physical spaces during informal and formal communications influenced the extent to which older patients participated in crucial decision‐making processes about their medications. Our observations revealed that health professionals' physical distancing from older patients at the bedside or their movements during ward rounds could be impeding potential opportunities for older patients to be involved in the discussions about their medications. Important changes to older patients' regular medications were usually made after ward rounds were completed. The consultant and junior medical staff moved to the private office spaces of the ward to have detailed discussions about each patient's medications by checking electronic notes. Formal interdisciplinary discussions about medications between senior and junior doctors largely happened away from potantial distractions at the bedside, and main focus of these discussions was to meet junior doctors' educational needs. However, this impeded a cooperative discourse where older patients or family members could contribute to medication decisions. This situation also maintained unequal power relations between patients and health professionals. In some cases, important decisions about patients' discharge medications were made during unplanned interactions between health professionals that occurred in ward corridors without the involvement of older patients. These unplanned communications tended to occur just before patients' discharge or during opportunistic situations when nurses or pharmacists encountered doctors in ward corridors and requested specific alterations in patients' medications.

These field notes in Table 4 provide a snapshot of a common pattern of how health professionals moved across different spaces when they communicated about older patients' medications during ward rounds.

**Table 4:** Field notes from an observation of ward rounds

‘The team included the registrar, the resident and the intern doctor stand in the ward corridor in front of computer to review the medications for a 79 years‐old patient on the eMAR. While standing in the corridor, the resident and registrar discuss the patient's inhaler technique at home and they conclude that the patient has poor technique. The resident suggests Handihaler® and summarises the medication changes to the registrar. Doctors move on the patient's room to talk about the patient's inhaler (Symbicort®‐budesonide and formoterol fumarate dihydrate). The registrar tells the patient that they will put her back on her normal dose that the patient used to take at home. The registrar informs the patient: “We'll put you back on what you were on before” and she changes the topic and starts talking about her bowels, explains the change of her laxatives to PRN (as needed) and that she can ask for it if she needs it. The ward round team leaves the patient's room, move to stand in the ward corridor outside the room and have further discussions about the patient's inhaler and aspirin’. (Med5_Obs_Subacute4_Interaction_26)

The observation displayed the complexity in ward round interactions, which stemmed from doctors' varied conversations in different spaces of the ward environment, and tended to limit patients' contribution in decision‐making. Institutional power of doctors was sustained during bedside visits because they controlled interactions during ward rounds. The patient's involvement was missing in the discussion related to her inhaler technique since this communication occurred away from bedside without the patient's presence. It can be associated with doctors' perception of the role of the pharmacist in educating the patient about the inhaler technique before discharge. During ward rounds, doctors were more inclined to involve older patients in medication decisions relating to symptoms that patients could actually feel such as constipation or pain. There was less involvement with patients for medication decisions related to ‘silent’ symptoms, such as blood pressure or cholesterol levels.

Along with health professionals' movement across different spaces, their use of spaces around the bedside area was important for older patients who had sensory deficiencies, such as sight impairment and hearing loss. In some cases, health professionals' lack of proximity to older patients impeded their engagement with the medication communication. During an interview, a 71‐year‐old patient reported that he was not fully able to comprehend the context of the medication communication if health professionals stood at a distance that prevented him from reading lips or hearing the conversation:

For me, it's important when I'm talking to the people that I'm looking at them, that I can see their face and their lips moving and that they're not standing two metres away but they're a bit closer… It's really quiet at the moment but give it another half an hour and there will be a lot of background noise … They might think, ‘*This guy's a dumb bastard’*, or, ‘*He's not listening to me’*, so I just back off and hope that my interpretation is right when I answer the question. (Pt37_Subacute2)

In addition to his sensory difficulties, the patient brought attention to external unpleasant distractions occurring in the bedside environment at specific times, which caused additional hindrance to his involvement in the interactions about medications with health professionals. The patient reported that he was disinclined to ask the same questions to health professionals multiple times, which led him to take on a passive role in the formal bedside interactions with health professionals.

#### 3.2.2 Utilization of mobile computer systems during formal patient encounters

Before the implementation of electronic medical records, the medication charts were in the public domain and placed at the foot of the beds, which could be viewed by patients and family members. The introduction of mobile computers to replace paper‐based medical records influenced the ways in which health professionals communicated with patients about medications at the bedside. During planned patient interactions, such as ward rounds or nurses' medication rounds, health professionals tended to place mobile computers at the end of the bedside or in the middle of the patient room, which impeded the free flow of the interaction about medications that could occur between health professionals and patients at the bedside. This issue was more frequently observed with nursing staff and doctors compared to pharmacists. Notably, when nurses administered medications, they were confronted with repetitious tasks that required concentrating on patients' eMARs on the screen, such as scanning barcodes of each medication, ticking the boxes as they put the medications in the medication cup and handing medications to patients in this cup. During one focus group, a 74‐year‐old patient and her husband expressed their reflections on how the medications were communicated by nurses during the medication administration processes (Table 5).

**Table 5:** A focus group conducted with the patient and the family member.

*Patient*: So they (nurses) don't say, ‘*Oh, here's your atorvastatin or here's your Plavix*® *(clopidogrel) and here's your spironolactone and here's your Vesicare*® *(solifenacin), and here's that, here's the other’*. They just bring in ‐ they've got it on their computer, and they mark it off, and then they give it to me to take. I guess seeing as they're careful about what they're looking at the medication out of the box compared to their computer screen, they're checking what they're giving me. So I have sort of trust in what's being given to me, in that regard.
*Family Member (Husband)*: I guess if you were having a little cup with nine or 10 tablets in, they are not going to stand there and say, ‘This is Endone®, and this is da‐da‐da’. *Excerpt Code: Pt7_FM5_FG7_WardMed2.

Although mobile computers provided nurses with easy access to medication details while in the bedside area, information about medications was seldom communicated to older patients themselves. Indeed, nurses controlled what information was shared with older patients during formal medication administration processes since there was no opportunity for patients or family members to access the information about medication changes or new medications prescribed following the patient's admission to the hospital. Nurses relayed medication information from computers to the patients selectively, where they only mentioned the name of a new medication or a recent change to patients' regular medications. The reasons behind medication changes were usually discussed amongst doctors in front of computers in private ward spaces where nurses' involvement was missing, which, in turn, led to limited information sharing from nurses to patients, even though patients attempted to be actively involved in medication communication at the bedside. Similarly, during a focus group, a family member of another older patient complained about computerization preventing her from checking on her mother's medications after transfer from an acute hospital to a subacute setting:

I noticed they don't have it, but they used to have the charts on the end of the bed, and I used to look through the chart to make sure that all the tablets are there. I think they now keep the charts somewhere else. (FM_Daughter_63F)

It was observed that the relocation of the medication information from paper medical records to the electronic systems put health professionals in a more privileged position since they can view all medication changes through computers when the formal patient encounter is taking place. Patients and family members no longer had access to which medications had been changed, ceased or newly prescribed since patients' admission to the hospital.

## 4 DISCUSSION

This study showed the dynamic complexities of how older patients' medications were managed across transitions of care through formal and informal modes of communication. Notes featured as reminders for both older patients and health professionals; however, they were used in different ways. Older patients' self‐created written notes represented a complementary communication tool enabling them to engage with health professionals to influence medication decisions during ward rounds or to ask for further clarification about the changes made to their medications across transitions of care. When older patients had difficulties in accessing health professionals to discuss medication issues, they created notes to remind themselves to raise these issues at the next encounter. Health professionals maintained brief notes under eMARs, which they strategically created to facilitate their formal interactions and also to prompt them to have spontaneous or planned interactions with older patients to check their medication preferences or concerns at the bedside. Electronic medical records created opportunities for health professionals to easily access and communicate important medication changes. However, use of the electronic medical records tended to reinforce health professionals' focus on addressing medication tasks rather than communicating with older patients.

These findings highlight the need to recognize the role of older patients' self‐created medication notes in compensating for communication that has been lost during care transitions and formal patient encounters. The use of these notes is underreported in the literature. Some studies supporting our findings have emphasized the benefits of patient‐held medication lists such as digital or printed structured paper tools that allow patients to carry and edit the lists of current medications when they were transferred between different health care settings.^26^, ^27^ According to these studies, patients perceived lists as a useful tool to mitigate the medication‐related information loss when they moved between different health care settings.^26^, ^27^ One particular study focused on a medication passport designed for personal use in which there were sections for completion by older patients such as current medications, changes to the medications and blank pages for the notes. Similar to our findings, older patients who created notes in these medication passports regarded them as an aide‐memoire, improving medication‐related communication with their general practitioners in the community^27^, ^28^ and health professionals at the hospital. However, patients also found that some health professionals did not really look at these notes even though patients carried these with them across care transitions.^27^

Our research has further identified that self‐created notes not only functioned as aide‐memoires but also empowered older patients to initiate opportunistic discussions with health professionals. However, these opportunistic discussions only occurred when health professionals came to the bedside and made themselves available to listen to older patients' concerns. As indicated by Liu et al.,^14^ collaborative discourse between older patients and health professionals in medication decisions was rarely observed during formal patient encounters, particularly at the point‐of‐care transitions, which can be explained by tensions between health professionals' organizational time commitments and prioritization of other care‐related activities. For shared decision‐making to occur, health care organizations should have systems in place to enhance partnerships between health professionals and patients during formal and informal encounters. Patients' involvement in medication decisions can be improved by providing patients with access to information designed to meet their individual needs and by providing materials facilitating information exchange between patients and health professionals.^29^ Structured paper or electronic tools such as tablets at the bedside can be used to empower older patients to create their own notes and write down their medication questions as they move between settings throughout their hospital stay. Health professionals need to be prompted to conduct regular formal visits with patients to clarify their medication concerns.

Prompts about older patients' medication management under eMARs encouraged pharmacists to initiate informal discussions with the patients with the purpose of examining their existing nonadherence or to confirm their preferences for managing medications following discharge. These observations built on Riegels et al.'s^30^ findings emphasizing that structured reminders embedded in electronic records actually helped physicians to initiate individualized conversations with the patients on ward rounds by encouraging them to talk about their concerns about their discharge plans and treatment regimens.^30^ Most of these prompts were created to meet institutional requirements to facilitate formal interactions amongst health professionals, but the ones that were created with inclusive language such as ‘Discuss outcomes with son’ or ‘Assess closer to discharge if patient happy to continue self‐management’ acted as directives for pharmacists to involve older patients or family members in decision‐making processes. Even though these notes promoted the informal communication between pharmacists and older patients or their family members, a multidisciplinary effort is needed to maximize the benefit of utilizing electronic records when communicating with older patients about their medications. Previous research highlighted that electronic medical records helped to improve medication safety and efficiency in record keeping across care transitions,^31^, ^32^, ^33^, ^34^ but they did not foster communication with patients. Instead, the presence of electronic medical records appeared to create tensions between completing electronic templates and meeting the needs of individual patients, which sometimes resulted in missing patients' narratives during communication encounters. Similarly, our observations revealed that opportunities were missed by nurses to create informal discussions with older patients about medications. Nurses were inclined to prioritize completion of documentation or clinical activities, over soliciting patients' preferences for medications. Nurses were able to access the same information as pharmacists during medication administration processes, with the prompts embedded in eMARs enhancing the informational continuity in relation to older patients' medications from admission to discharge. However, it was obvious that the demarcations of health professionals' roles in communicating about medications with patients led to their selective use during formal patient encounters. Therefore, health professionals, particularly nurses and doctors, could use these prompts as conduits to initiate informal communication with older patients to ensure their understanding of the medications that were altered since admission and to seek out their preferences for medication management following discharge.

Older patients' involvement in the medication communication was influenced by health professionals' spatial behaviour and the utilization of the computers during their formal and informal patient encounters. As previously highlighted, doctors' flexibility of moving across different spaces when making important decisions about medications created a hindrance to older patients' potential input, which contributed to maintenance of medical dominance in decision‐making processes.^35^ Although computers on wheels provided health professionals with an opportunity to access and communicate the important medication changes to the older patients at the bedside well before their discharge, the limited space in the patients' rooms created additional challenges to having comprehensive medication discussions. Our observations were consistent with the past studies where the structure of ward rounds involved the team surrounding the patient's bedside with a junior doctor responsible for typing the notes on computers at the end of the bed and a consultant controlling the interactions according to a preset agenda.^36^, ^37^ Older patients' sensory deficiencies and interruptions in the patient rooms were rarely scrutinized by health professionals when organizing their physical positioning or distance to the patients during structured ward rounds. This strengthened the unequal power distribution between patients and providers, where most older patients remained content with the limited amount of medication information and participated only when they were invited to communicate. Prior research also indicated that the use of computers constituted a third agent leading to communication breakdowns during patient encounter,^38^, ^39^ which we also noted when nurses determined what medication information can be shared with older patients and family members during medication rounds. Therefore, simplified information about medication changes should be made available for older patients and family members through user‐friendly tablet computers or patient portals at the bedside so that they can track any changes following their hospitalization and also access written information simultaneously during ward rounds or nurses' medication rounds.

This study has limitations. Potential observer effects may have occurred with participating health professionals when they were communicating with patients. However, observer effects were mitigated by building rapport with health professionals and continuous observations over several weeks in a single ward before moving to the next ward. Additionally, the study was conducted at two metropolitan hospitals, and therefore, the findings may not be transferable to individuals located in regional or rural areas. More nurses than doctors and pharmacists participated in observations within this study, as they were the health professionals most commonly present at the bedside.

## 5 CONCLUSIONS

This study illustrated the importance of older patients' self‐created medication notes not only as an aide‐memoire but also as a tool enabling them to initiate informal communication with health professionals to seek out further clarification about their medications when they move between different settings. These notes signalled older patients' motivation to take on an active role in medication decisions. Formal prompts embedded in the eMAR regarding older patients' medications acted as useful cues for health professionals to have face‐to‐face discussions with patients on specific medications, and to clarify older patients' preferences of managing their medications after discharge. However, prompts were mostly used by pharmacists rather than medical or nursing staff. Environmental influences within hospital settings limited older patients' potential engagement with informal and formal medication interactions and decision‐making processes. Further investigations are required on how to address the environmental struggles affecting medications decisions, disparities in accessing electronic medication information between health professionals and older patients during formal encounters, and distractions and interruptions occurring at bedside spaces.

## AUTHOR CONTRIBUTIONS

Guncag Ozavci and Elizabeth Manias made substantial contributions towards the design and drafting of the manuscript. Guncag Ozavci and Elizabeth Manias were responsible for data collection/analysis and data interpretation. Elizabeth Manias, Tracey Bucknall, Christine Jorm, Carmel Hughes and Robyn Woodward‐Kron made critical revisions to the paper for important intellectual content. Elizabeth Manias, Tracey Bucknall, Christine Jorm, Carmel Hughes and Robyn Woodward‐Kron obtained funding. All authors have agreed on the final version of the review.

## CONFLICTS OF INTEREST

The authors declare that there are no conflicts of interest or any employment or prior relationship with the institutions and participants.

## Supporting information

Supporting information.

## Data Availability

The data that support the findings of this study are available from the corresponding author upon reasonable request.
