# Supplementary material for: Creating opportunities for patient participation in managing medications across transitions of care through formal and informal modes of communication
Source: Health Expect. 2022 May 27;25(4):1807–20. doi: 10.1111/hex.13524 (PMC9327867; doi:10.1111/hex.13524)
Supplement: Supplementary file 1 — Supporting information. [file HEX-25--s001.docx]

**Individual interviews with patients**

1. Can you tell me about the medications you have been taking at home (what, when, what for, who gives them to you)? Have your medications changed while you have been in hospital? If so, what are these new medications (what, when, what for, who gives them to you)?
2. Were your medications changed when you first came to hospital? How involved did you feel about decisions made regarding changes to your medications when you first came into hospital?
3. What things do you think are important to discuss about your medications when you come into hospital?
4. What things do you think are important to discuss about your medications when you leave the hospital?
5. Have you transferred from another ward? If so, what were you told about your medications before you moved to this ward?
6. How could we improve the information that you get about your medications when you move from one place to another?
